# Geographic Atrophy in Age-Related Macular Degeneration Is Associated With Distinct T-Cell Differentiation Profile

**DOI:** 10.1167/iovs.67.13.3

**Published:** 2026-11-02

**Authors:** Anja Blaabjerg Hartelius, Alexander Kai Thomsen, Yousif Subhi, Torben Lykke Sørensen, Marie Krogh Nielsen

**Affiliations:** 1 Department of Ophthalmology, Zealand University Hospital, Roskilde, Denmark; 2 Department of Ophthalmology, Rigshospitalet, Glostrup, Denmark; 3 Department of Clinical Medicine, University of Copenhagen, Copenhagen, Denmark; 4 Department of Ophthalmology, University Hospital of Southern Denmark, Sønderborg, Denmark; 5 Department of Regional Health Research, University of Southern Denmark, Odense, Denmark

**Keywords:** AMD, geographic atrophy, neovascular AMD, inflammation, T cells

## Abstract

**Purpose:**

Increasing age is the strongest risk factor in developing AMD. Growing evidence suggests that systemic immunosenescence is implicated in its pathogenesis. This study aims to characterize age-related T-cell differentiation in late-stage AMD by comparing patients with geographic atrophy (GA) and neovascular AMD (nAMD) to a shared healthy control group.

**Methods:**

Patients with GA or nAMD were recruited from an outpatient AMD-clinic. Individuals with immunological disease, cancer or use of immune-modulating medication were excluded. Blood samples from all participants were stained with monoclonal antibodies and analyzed with flow cytometry to assess T-cell subsets (naive, central memory [CM], effector memory [EM], and effector memory with CD45ra+ [EMRA]) and expression of the age-associated costimulatory markers (CD27, CD28, and CD56).

**Results:**

One hundred twenty-eight patients were included in this study. Patients with GA demonstrated significantly higher levels of CD8+CD28-EM T cells (*P* = 0.038) and significantly lower levels of CD4+CD56+CM T cells (*P* = 0.021) compared with healthy controls. Patients with nAMD showed significantly higher levels of naive CD4+CD56+ and CD4+CD56+CM T cells (*P* = 0.008 and *P* = 0.008) compared to patients with GA.

**Conclusions:**

Patients with GA demonstrated an increased proportion of CD8+CD28− EM T cells, a finding similar to other infectious, autoimmune, and neurodegenerative disorders. The increased expression of CD56+ T cells in patients with nAMD supports CD56 having a potential role in angiogenic regulation. Contrarily, we suggest the reduced CD56 expression in patients with GA might contribute to GA pathogenesis.

AMD is a leading cause of irreversible vision loss in the elderly.^1^^–^^5^ Despite abundant research, its pathogenesis remains incompletely understood. Late-stage disease manifests as neovascular AMD (nAMD), driven by choroidal neovascularization, and geographic atrophy (GA), defined by progressive atrophy of the choriocapillaris, retinal pigment epithelium and photoreceptors.

Intravitreal anti-VEGF therapy has substantially improved outcomes in nAMD, effectively limiting vision loss in most patients.^6^ Until the recent introduction of Pegcetacoplan GA has had no treatment options. The intravitreal complement C3 inhibitor has demonstrated efficacy in slowing GA progression.^7^^–^^9^ The drug is approved by the United States Food and Drug Administration^7^ but the European Medicines Agency declined marketing authorization in 2024.^10^ Given the variability in treatment response in nAMD and the absence of approved therapies for GA in Europe, an understanding of the causes of nAMD and GA and what drive their progression becomes vital for future interventions.

Age is a major risk factor for both nAMD and GA,^11^^–^^15^ and increasing evidence indicates a role of systemic immune dysregulation in disease pathogenesis.^5^^,^^13^^,^^16^^–^^30^ Aging is accompanied by immunosenescence,^31^^,^^32^ and particularly the adaptive immune system undergoes profound age-dependent changes.^32^

Age-associated T-cell change is characterized by a decline in naive T cells and an expansion of central memory (CM) and effector memory (EM) T cells.^33^ Thus the immune system loses naivety and gains differentiation and cytotoxic properties with age. These alterations may influence retinal immune homeostasis, VEGF-expression on injury and the promotion of angiogenesis. Thus they are perceived to be implicated in AMD pathogenesis.^5^^,^^13^^,^^14^^,^^34^^–^^37^

Our group has previously shown that age-related T-cell differentiation may contribute to late-stage AMD.^13^^,^^14^^,^^28^^,^^34^^,^^37^ It is in extension of these results that this study focuses on specific phenotypic age-related differentiations in CD4+ and CD8+ T cells.

Age-related T-cell differentiation includes the gradual loss of the costimulatory CD28 and CD27 receptors on CD4+ and CD8+ T cells. CD4+ T cells lose expression of CD27 before CD28, whereas CD8+ T cells show the opposite pattern.^32^ In young healthy individuals CD28− T cells are relatively low in numbers, but particularly the CD8+CD28− subset increases with age.^38^^,^^39^

Studies have found increased expression of CD56+ on CD8+ T cells in patients with nAMD.^13^^,^^14^ CD56 is another age-associated costimulatory marker and its expression on T cells is associated with increased cytotoxicity.^31^ CD56 expression is known to increase with T-cell differentiation and can be expressed on naive, CM, and EM T cells. Increased CD56 expression on these cells is considered a hallmark of T-cell aging.^24^^,^^28^^,^^31^^,^^40^

A senescent and cytotoxic immune system is further characterized by accumulation of terminally differentiated effector memory T cells expressing CD45ra (EMRA). These cells exhibit enhanced cytotoxicity and features of natural killer (NK) cells including upregulation of CD56.^31^

As the abovementioned changes previously have been described among patients with AMD^13^^,^^14^^,^^24^^,^^32^ this study seeks to investigate and map the potential loss of CD27 and CD28, the potential increase in CM, EM and EMRA T cells, and the potential increase of CD56 expression on CD4+ and CD8+ T cells in patients with nAMD and GA and compare the findings to the same healthy control group.

## Methods

### Study Design and Participants

This study was conducted at the Department of Ophthalmology, Zealand University Hospital, Roskilde, Denmark and was approved by the Regional Committee of Ethics in Research of the Region of Zealand (SJ-385). The study was conducted in accordance with the Declaration of Helsinki. Verbal and written informed consent were obtained from all participants prior to inclusion.

The participants were recruited from the retinal outpatient clinic at the Department of Ophthalmology, Zealand University Hospital, Roskilde, Denmark. Patients with GA or nAMD who had not previously received any type of intraocular anti-VEGF treatment were included. Participants were excluded if they had any retinal disease other than GA or nAMD, reported acute illness within the preceding five days, had a history of or current inflammatory disorder or cancer, or had or were receiving any immunomodulating therapy (e.g. methotrexate, corticosteroids in any form). Participants with CRP > 15 were excluded from the study post hoc to minimize the influence of acute systemic inflammation. To closely match the healthy control group with the same age and environmental and socioeconomic factors as the patients with GA and nAMD, healthy and biologically unrelated partners were invited to participate in the study as healthy controls.

All participants were interviewed about their lifestyle, medical history, and current medication. Body mass index was calculated by using self-reported weight and height. Smoking status was categorized as current, former or never. Current smokers had smoked within the past year regardless of cessation attempts. Former and never smokers had not smoked within the past year. Former smokers had a lifetime consumption of >100 cigarettes (five packs), whereas never smokers had smoked <100 cigarettes. Information about exercise was assessed using a single-sentence question validated for observational studies that has previously been validated on patients with nAMD.^5^^,^^36^ Cardiovascular disease was defined as clinically diagnosed coronary, cerebrovascular, or peripheral arterial disease, heart failure, or cardiac arrhythmia. The cardiovascular risk factors hypertension, hypercholesterolemia and type 2 diabetes were recorded separately.

### Retinal Diagnosis and Grading of GA

All participants were thoroughly clinically examined by a retinal specialist using bilateral ophthalmoscopy with a 90-diopter lens to visualize retina in mydriasis. Digital color fundus photography (Carl Zeiss, Jena, Germany), Spectral-Domain Optical Coherence Tomography and fundus autofluorescence imaging (Spectralis HRA-OCT, SLO Heidelberg Engineering, Heidelberg, Germany) were performed on all participants, and those with presumed nAMD were additionally examined with both fluorescein and indocyanine green angiography.

The participants were categorized into subgroups of AMD using the Clinical Age-Related Maculopathy Staging (CARMS) system, which classifies AMD across five stages and categorizes progression from early changes and intermediate drusen to GA and nAMD.^41^ The healthy controls had a normal macula with 0–10 small drusen and no pigment abnormalities (corresponding to CARMS grade 1). Early AMD (CARMS grade 2) was defined as ≥ 10 small drusen, <15 intermediate drusen or pigment abnormalities (hyperpigmentation or hypopigmentation), whereas intermediate AMD (CARMS grade 3) was defined as ≥15 intermediate drusen or any large drusen. Patients with sharply demarcated areas of atrophy in the macular area, involving the RPE and outer photoreceptor layer (corresponding to CARMS grade 4) were categorized as having GA. Patients with nondrusenoid pigment epithelial detachments, serous retinal detachments, choroidal neovascular membrane with subretinal or sub-RPE hemorrhages or subretinal fibroses (corresponding to CARMS grade 5) were categorized as having nAMD. Because both eyes were examined, it was decided that if the CARMS stages or the GA pattern type differed between the eyes, the eye with the more advanced CARMS stage was chosen to represent the participant. Fellow eye diagnoses are presented in Table 1.

**Table 1.** Fellow Eye Diagnoses in Study Population

	Fellow Eye Diagnosis		
Study Eye Diagnosis	Early to Intermediate AMD (CARMS 2–3)	GA (CARMS 4)	nAMD (CARMS 5)
GA (CARMS 4)	2 (4.8%)	40 (95.2%)	NA
nAMD (CARMS 5)	14 (32.6%)	5 (11.6%)	24 (55.8%)

### Blood Sampling and Flow Cytometry

Each participant had two blood samples drawn from the antecubital vein: One 5 mL vacutainer tube containing ethylenediaminetetraacetic acid coagulant for flow cytometry and one 3 mL vacutainer tube coated with lithiumheparin for determining CRP. All analyses were initiated within four hours of blood sampling.

Flow cytometry was used to quantify cell morphology, size, granularity and surface protein expression in each blood sample. This was done by first performing a white blood cell count (Sysmex KX-21N; Sysmex Corporation, Kobe, Japan) that calculated a blood volume equivalent to 5 × 10^5^ leukocytes from each participant. The red blood cells in these blood volumes were then lysed in a 50 mL tube by adding a red blood cell lysis buffer (Nordic Biosite AB, Täby, Sweden) and then incubated for 10 minutes in the dark room at room temperature. The cells were then washed three times by spinning the tubes in a centrifuge for five minutes at 500*g*, decanting the supernatant and then resuspending the blood in isotonic buffer (IsoFlow Sheath Fluid; Beckman Coulter Inc., Brea, CA, USA). The following four monoclonal antibodies were then added to the isolated leukocytes (Supplementary Table S1): Phycoerythrin-CY7 CD14 (IgG2a κ, Clone M5E2, catalog no. 301814; Biolegend, San Diego, CA, USA), Pacific Blue CD16 (IgG1 κ, Clone 3G8, catalog no 302032; Biolegend), Brilliant Violet 510 HLA-DR (IgG2a κ, Clone L243 catalog no. 307646; Biolegend), and Phycoerythrin CD200 (IgG1 κ, Clone 325516, catalog no. FAB27241P; R&D Systems Inc., Minneapolis, MN, USA).

To create a negative control, a separate identical tube of leukocytes was exposed to the following fluorochrome-matched negative isotypes: Phycoerythrin CY7 IgG2a κ (Clone MOPC-173, catalog no. 400232; Biolegend), Pacific Blue IgG1 κ (Clone MOPC-21, catalog no. 400151; Biolegend), Brilliant Violet 510 (IgG2a κ, Clone MOPC-173, catalog no. 400268; Biolegend), and Phycoerythrin IgG1 κ (Clone MOPC-21, catalog no. 555749; BD Biosciences, San Jose, CA, USA). The negative control tubes were also incubated the dark at room temperature for 20 minutes as recommended by the manufacturer. Afterward the cells were washed, resuspended in isotonic buffer and immediately analyzed using flow cytometry (BD FACSCanto II System; BD Biosciences).

### Gating Strategies

Singlet leukocytes were identified on a forward scatter area-forward scatter height plot. These leukocytes were then categorized as granulocytes, monocytes and lymphocytes by gating the cells on a forward-side scatter plot. The lymphocytes were then gated and separated into two main groups by their expression of CD4+ or CD8+ (Fig. 1) to identify CD4+ and CD8+ T cells. Both CD4+ and CD8+ cells were additionally gated and isolated for their expression of CD45ra, CD45ro and CCR7 which resulted in two diagrams representing respectively CD4+ and CD8+ with their naive (CD45ra+CD45ro-CCR7+), central memory (CD45ra−CD45ro+CCR7+), effector memory (CD45ra−CD45ro+CCR7−) and EMRA (CD45ra+CD45ro+CCR7−) markers (Fig. 1). And finally, to classify the T cells for their costimulatory markers T-cell subsets were gated for CD27, CD28, and CD56 (Supplementary Fig. S1).

**Figure 1.**
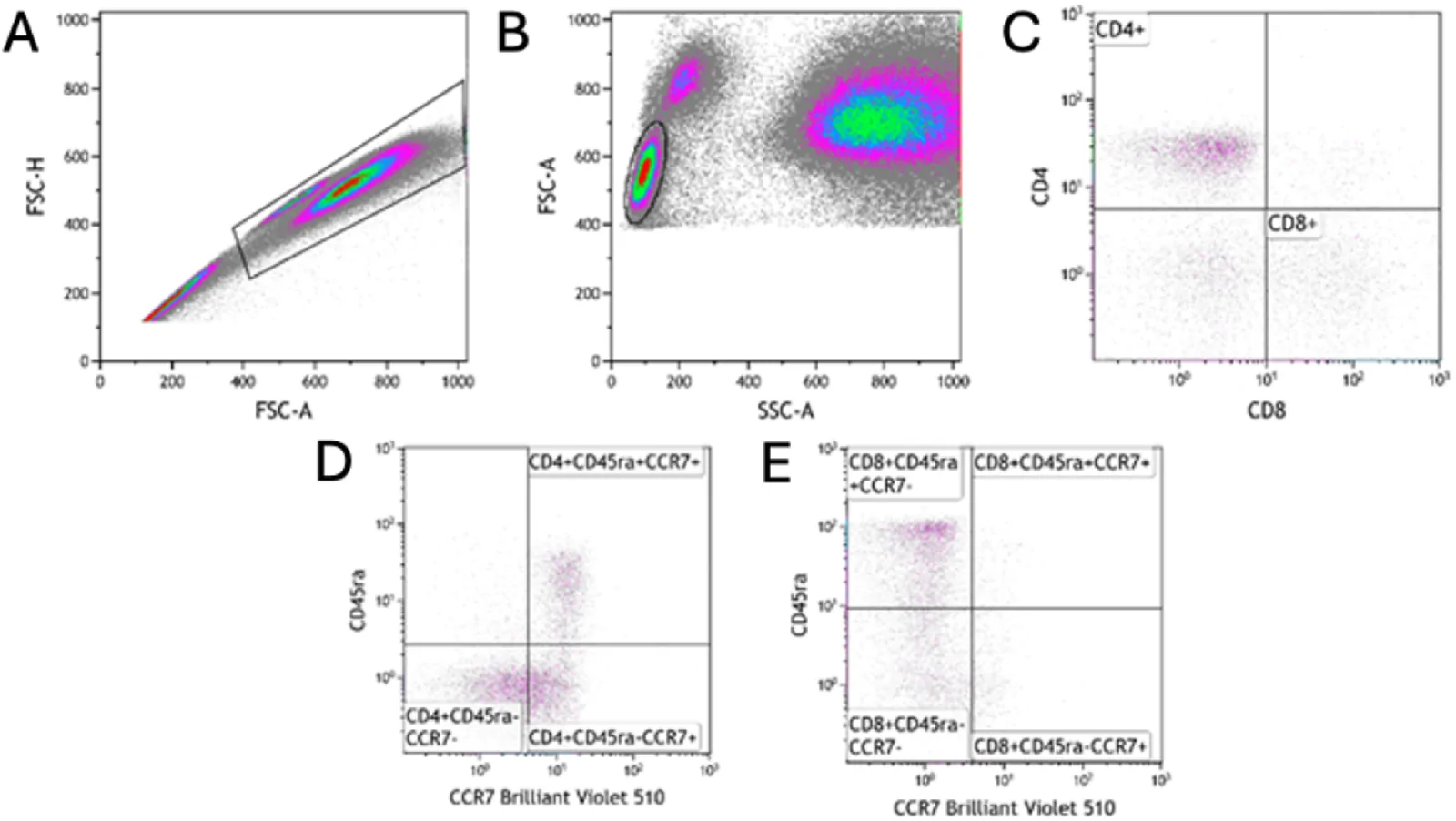
Gating strategy**:** singlet leukocytes were identified **(A)**, and lymphocytes were isolated from monocytes and granulocytes **(B)**. The lymphocytes were gated for CD4+ and CD8+ expression **(C)** and the cells in these two subgroups were then gated for expression of functional markers for naive, CM, EM, and EMRA T cells (**D**, **E**). Cells expressing CD45ra+CD45ro−CCR7+ were considered naive, CD45ra−CD45ro+CCR7 were considered CM, CD45ra−CD45ro+CCR7− were considered effector memory T cells (EM), CD45ra+CD45ro+CCR7− were considered EMRA. FSC-H, forward scatter height; FSC-A, forward scatter area; SSC-A, Side scatter area.

### Data Analyses and Statistics

All data from the flow cytometry was analyzed using Kaluza Analysis (version 1.5.20.365.16139; Kaluza Analysis, Brea, CA, USA). Analysis was performed by two individual investigators (author MKN and YS) who were blinded from diagnosis and any clinical information. The participants’ demographic characteristics, lifestyle and comorbidities were compared across the three groups (healthy controls, GA and nAMD). See Table 2 for specific test methods.

**Table 2.** Participant Characteristics

	Diagnosis			
	Healthy, *n* = 43	GA, *n* = 42	nAMD, *n* = 43	*P* Value
Age, mean (SD)	76.4 (5.5)	77.64 (6.5)	77.7 (4.9)	0.210*
Female sex	22 (51.2%)	27 (64.3%)	25 (58.1%)	0.495^†^
Body mass index, mean (SD)	26.4 (4.0)	26.2 (5.8)	25.6 (3.8)	0.720^‡^
Smoking status				0.012^†^
Current	9 (21.4%)	1 (2.3%)	7 (16.3%)	
Previous	21 (50.0%)	16 (37.2%)	20 (46.5%)	
Never	12 (28.6%)	26 (60.5%)	16 (37.2%)	
Daily exercise	31 (72.1%)	31 (73.8%)	30 (69.75)	0.969^†^
Hypertension	12 (27.9%)	20 (47.6%)	18 (41.95)	0.156^†^
Hypercholersterolemia	10 (23.3%)	10 (23.8%)	11 (25.6%)	0.966^†^
Cardiovascular disease	5 (11.6%)	19 (45.2%)	5 (11.6%)	<0.001^†^
Type 2 diabetes	1 (2.3%)	5 (11.9%)	3 (7.0%)	0.193^†^

* Kruskal-Wallis test.

† Fisher's exact test.

‡ One-way ANOVA.

Statistical analysis was performed using the R software version 4.2.3 (R Foundation for Statical Computing, Vienna, Austria). Demographic normally distributed data was presented as mean and SD, comparisons were tested with ANOVA. Demographic not normally distributed data was presented as median and interquartile range, and comparisons were made using non-parametrical tests. Categorical data was presented in percentages and tested using Fisher's exact test. Proportions of T cells were logarithmically transformed to fit assumption of normality and were compared between groups with analysis of covariance, adjusted for age as this influences these proportions, as well as smoking status and cardiovascular disease as these were significantly different between groups. A *P*-value < 0.05 was interpreted as statistically significant. Post hoc false discovery rate correction was made on all statistically significant *P*-values.

## Results

### Participant Characteristics

A total of 128 patients were included in this study of which 43 participants had nAMD, 42 participants had GA and 43 participants were healthy controls (Table 2). There was no significant age difference between the groups (Kruskal-Wallis test, *P* = 0.210) nor between body mass index, sex, daily exercise, hypertension, hypercholesterolemia or diabetes.

The participants differed significantly on cardiovascular disease and smoking status (Table 2). The GA group had a higher prevalence of cardiovascular disease (*P* < 0.001) and a lower smoking prevalence (*P* = 0.012) when compared to the nAMD group and healthy controls.

### CD4+ and CD8+ T-Cell Differentiation

The distribution of naive, CM, EM and EMRA among the CD4+ and CD8+ T cells are illustrated in the circle diagrams for the healthy control group, GA and nAMD respectively (Fig. 2).

**Figure 2.**
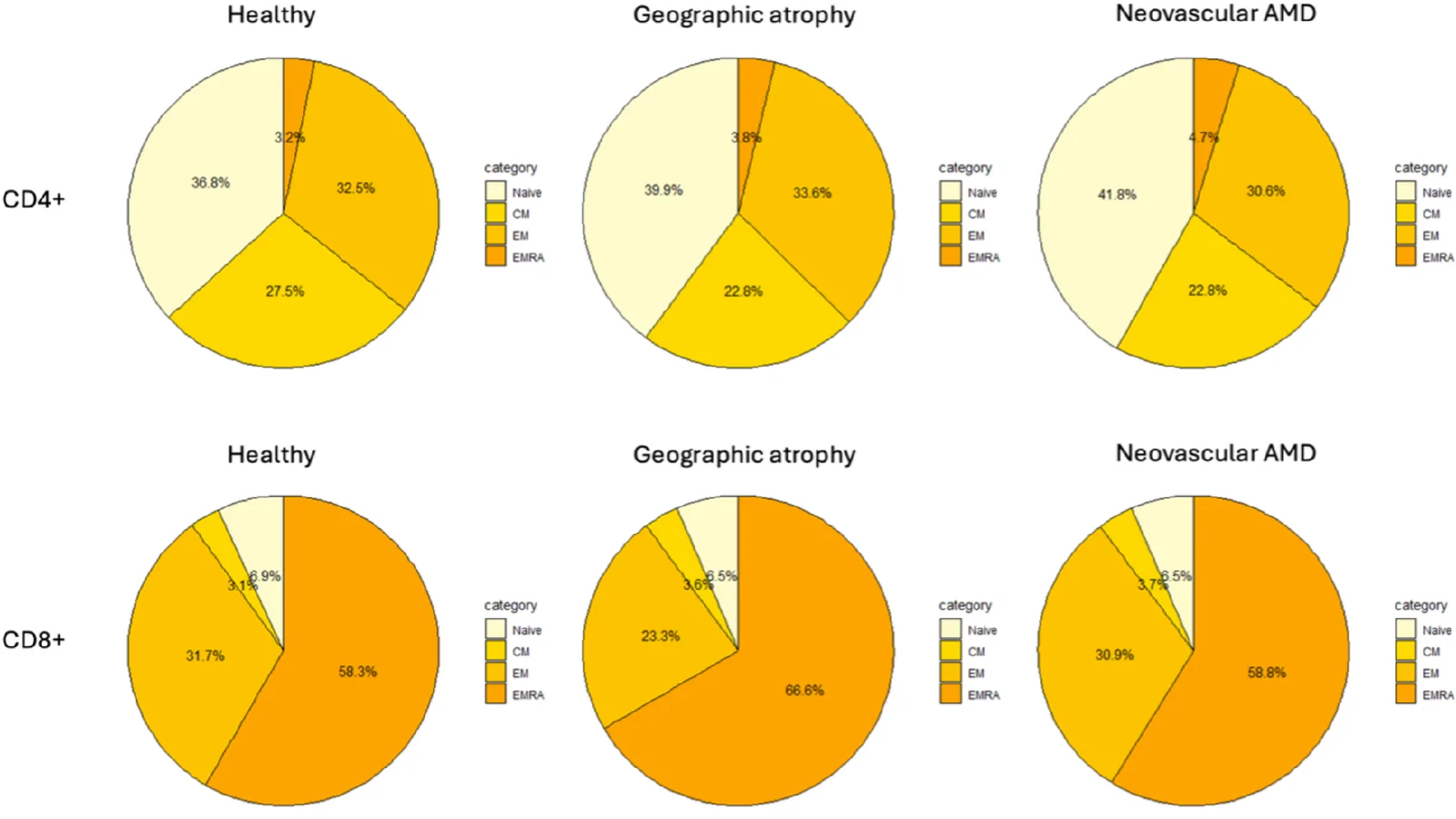
Surface marker distribution across the three participant groups. We found no significant differences among the distribution of the functional surface markers between the patients with GA, nAMD and healthy controls (*P* > 0.05, Fisher's exact test).

The distribution of CD4+ naive, CM, EM and EMRA T cells showed no statistically significant differences between the healthy controls, GA and nAMD. Neither were any significant differences between the CD8+ naive, CM, EM, and EMRA T cells found when comparing the three groups.

### Age Marker Comparisons

The T-cell age markers were compared between all three groups (Table 3). Patients with GA had a significantly lower level of CD4+CD56+CM T cells when compared to both the healthy controls (*P* = 0.021) and nAMD (*P* = 0.008). Patients with GA also had a significantly higher level of CD8+CD28−EM T cells when compared to the healthy controls (*P* = 0.038).

**Table 3.** Age Marker Comparisons

				*P* Value*		
	HC	GA	nAMD	GA Vs. HC	nAMD Vs. HC	GA Vs. nAMD
CD4+ T cells						
Naive CD27−	69.83 (20.15)	68.96 (15.88)	63.31 (17.03)	0.841	0.832	0.710
CM CD27−	76.05 (15.27)	80.52 (12.22)	80.39 (13.43)	0.346	0.129	0.801
EM CD27−	77.19 (8.41)	85.17 (9.71)	80.95 (10.01)	0.100	0.407	0.096
EMRA CD27−	98.98 (0.95)	99.44 (0.99)	98.95 (0.93)	0.115	0.252	0.298
Naive CD28−	69.84 (20.14)	69.24 (21.80)	63.31 (17.02)	0.182	0.692	0.097
CM CD28−	75.37 (15.58)	80.18 (14.37)	80.10 (14.32)	0.116	0.191	0.567
EM CD28−	71.77 (8.52)	83.37 (11.01)	78.57 (14.44)	0.050	0.523	0.395
EMRA CD28−	98.75 (1.13)	99.22 (0.65)	98.78 (1.19)	0.053	0.305	0.141
Naive CD56+	0.03 (0.04)	0.00 (0.01)	0.06 (0.10)	0.059	0.068	**0.008**
CM CD56+	0.23 (0.22)	0.06 (0.22)	0.32 (0.28)	**0.021**	0.564	**0.008**
EM CD56+	0.32 (0.42)	0.31 (0.60)	0.55 (0.42)	0.175	0.786	0.093
EMRA CD56+	0.03 (0.14)	0.02 (0.42)	0.02 (0.52)	0.521	0.808	0.613
CD8+ T cells						
Naive CD27−	94.13 (6.43)	94.91 (5.69)	96.67 (2.87)	0.771	0.991	0.820
CM CD27−	97.72 (1.72)	97.51 (2.03)	98.14 (3.69)	0.168	0.986	0.138
EM CD27−	79.52 (11.10)	88.91 (7.45)	85.67 (10.33)	0.114	0.250	0.134
EMRA CD27−	88.8 (5.34)	90.08 (8.36)	91.53 (6.85)	0.868	0.112	0.280
Naive CD28−	94.19 (6.40)	96.79 (5.04)	96.69 (2.78)	0.876	0.862	0.760
CM CD28−	97.71 (1.73)	97.56 (2.26)	98.15 (3.74)	0.205	0.959	0.182
EM CD28−	81.16 (9.96)	90 (8.52)	84.49 (14.20)	**0.038**	0.425	0.213
EMRA CD28−	94.55 (4.53)	95.94 (3.28)	95.36 (2.93)	0.285	0.102	0.890
Naive CD56+	94.17 (6.42)	96.78 (6.14)	96.64 (3.04)	0.978	0.924	0.917
CM CD56+	97.75 (1.41)	98.04 (1.76)	98.13 (3.68)	0.240	0.933	0.227
EM CD56+	77.59 (11.14)	86.86 (16.18)	76.43 (18.50)	0.103	0.748	0.128
EMRA CD56+	57.04 (21.74)	44.23 (21.19)	63.16 (32.01)	0.553	0.908	0.580

HC, healthy controls.

Bold values indicate statistical significance (*P* < 0.05).

* Analysis of covariance on logarithmically transformed data adjusted for age, smoking status, and cardiovascular disease with post-hoc false discovery rate corrections on statistically significant results.

Patients with GA had a significantly lower level of naive CD4+CD56+ T cells when compared to nAMD (*P* = 0.008). GA and nAMD patients tended to have lower levels of naive CD4+CD56+ T cells compared to healthy controls, although not statistically significant (*P* = 0.059 and *P* = 0.068, respectively).

Patients with GA also had a tendency of having a higher level of CD4+CD28-EM T cells and CD4+CD28-EMRA T cells when compared to healthy controls, although not statistically significant (*P* = 0.050 and *P* = 0.053, respectively) When comparing the patients with nAMD to the healthy controls no significant differences in the T-cell age markers were found.

### Fellow Eye Diagnoses

Fellow eye diagnoses are presented in Table 1. GA was bilateral in 40 (95.2%) patients, whereas two (4.8%) had early to intermediate AMD (CARMS 2–3) in the fellow eye. Among patients with nAMD 24 (55.8%) had bilateral nAMD, 14 (32.6%) had early to intermediate AMD and five (11.6%) patients had GA in the fellow eye.

## Discussion

This study sought to investigate whether there were any differences in the age-related T-cell differentiations when comparing patients with GA and nAMD to healthy controls. We observed a significantly higher level of CD8+CD28−EM T cells in patients with GA compared to healthy controls. This finding is consistent with previous reports suggesting CD8+ T cells tend to lose CD28 expression before CD27 because they differentiate with age, whereas CD4+ T cells typically exhibit the reverse progression.^32^ We also observed an overall increase in the CD8+CD28− T-cell subtypes in both the GA and nAMD groups when compared to the healthy controls. Although not statistically significant, the trend supports the hypothesis that CD8+ T cells may undergo more profound age-related phenotypic changes than CD4+ T cells.^28^^,^^38^^,^^39^^,^^42^^–^^44^ These results further implicate immune aging, particularly within the CD8+ compartment, as a potential contributing factor in the pathogenesis of GA and possibly nAMD.

The significantly higher level of CD8+CD28−EM T cells in patients with GA may be a distinct immunological feature of GA that, to our knowledge, not yet has been described in AMD pathology. Accumulation of CD8+CD28− T cells has been observed in HIV positive individuals^38^ and is perceived to indicate higher risk of mortality in acute COVID-19 infections.^38^^,^^45^ The accumulation is also described to reflect the severity of CMV infection, alongside elevated levels of CD4+CD28-.^38^^,^^46^ Thus CD8+CD28− T-cell accumulation is evident in infectious and autoimmune disorders.^38^

The loss of CD28 among CD8+ T cells is also seen in neurological disorders, including amyotrophic lateral sclerosis^47^ and susac syndrome.^48^ In the neurodegenerative Alzheimer's disease clonal expansion of CD8+CD28− T cells is observed in both peripheral blood, cerebrospinal fluid (CSF) and in the brain. In Alzheimer's disease the CD8+CD28− T cell level shows a negative correlation with cognitive function.^49^^–^^51^ Taken together, the broad implication of CD8+CD28− T cells in infectious, autoimmune, and neurodegenerative conditions suggests that their accelerated accumulation in patients with GA may reflect a deeper neuroimmune dysregulation.

Previous studies have associated CD56+ T cells with AMD. Faber et al.^13^ reported increased CD56+CD28− T cells in AMD overall, whereas Subhi et al.^14^ specifically found increased CD56 expression among the CD8+ T cells in nAMD. In this study, CD56+ T cells did not differ significantly between patients with nAMD and healthy controls. Instead, patients with GA had significantly lower levels of CD4+CD56+CM T cells when compared to both nAMD and healthy controls. Thus our findings do not demonstrate increased CD56 expression in nAMD but suggest that the previously reported increased CD56 expression in late AMD subtypes may be less pronounced in GA. Faber et al.^13^ focused on CD28- T cells, whereas we did not further stratify CD4+CD56+CM T cells by CD28 status, which is why the comparison should be done with caution.

CD56 is a well-established marker of immunosenescence and a classic surface antigen for NK cells. It is also expressed by various other cytokine-producing and cytotoxic immune cells, including α and β T cells.^31^ In cultured RPE cells, CD56+ T cells have been shown to secrete IFN-γ,^52^ a cytokine known to induce inflammatory stress responses that promote angiogenesis.^17^ This IFN-γ secretion stimulated VEGF production in the RPE cells,^52^ and a separate human study confirmed that IFN-γ and IL-1β increase VEGF expression in RPE cells.^53^ The resulting proangiogenic microenvironment at the RPE barrier facilitates choroidal endothelial cell migration and proliferation, contributing to the neovascularization characteristic of nAMD^54^ ultimately implicating CD56+ T cells in the regulation of angiogenic processes.

CD56+ uterine NK cells are also recognized as key producers of angiogenic factors in the endometrium during the peri-implantation period.^55^^,^^56^ Similar mechanisms have been observed in renal cell carcinoma, in which infiltrating CD56+ NK cells exhibit a shift toward a non-classical, decidual-like phenotype, characterized by reduced cytotoxicity and enhanced proangiogenic activity in tumor tissue.^57^ This may suggest that CD56+ cells may have a broader role in promoting angiogenesis beyond the ocular context.

Our group has previously found an increased number of CD8+CD56+ T cells in patients with nAMD.^14^ In this study CD4+CD56+CM T cells were significantly higher among patients with nAMD when compared to patients with GA, whereas no significant difference was observed between nAMD and healthy controls. Together with previous findings these AMD-subtype differences make us hypothesize that CD56+ expression may be related to the angiogenic processes that are seen in nAMD pathology.

In contrast we also hypothesize that the lower CD56+ T cell levels observed in GA may represent a distinct feature of this AMD subtype. Additional data presented in this study support this direction as patients with GA tended to have lower levels of naive CD4+CD56+, CM, EM, and EMRA T cells. Compared with healthy controls, this difference reached statistical significance for CD4+CD56+ CM T cells, whereas naive CD4+CD56+ and CD4+CD56+CM T cells were significantly lower compared with nAMD. This is interesting because CD4+ T-helper cells are known to release cytokines such as TNF-α and IFN-γ,^58^ and TNF-α is thought to mediate macular neovascularization and increase expression of VEGF.^59^ We propose that the observed trend toward lower levels of the abovementioned CD4+CD56+ T-cell subtypes among patients with GA may reflect reduced angiogenic activity relative to nAMD, potentially linked to diminished CD4+ and CD56+ expression. However, this interpretation remains speculative because not all differences reached statistically significance.

Patients with GA had a higher prevalence of CVD but a lower prevalence of smoking than patients with nAMD and healthy controls. Because smoking is an established risk factor for AMD^60^^–^^62^ and both smoking^63^^,^^64^ and CVD^65^^,^^66^ have been associated with alterations in T-cell phenotypes, these group differences may have influenced the observed immune profiles. The lower smoking prevalence in the GA group is unlikely to fully explain our results and may, if anything, have attenuated the age-related T-cell differentiation. In contrast, the higher CVD prevalence may have contributed as CD8+CD28− T-cell accumulation in particular has been associated with CVD.^38^ Although both factors were included as covariates in the statistical analyses, residual confounding cannot be excluded.

The fellow eye distribution in our cohort was notable with 95.2% of patients with GA having bilateral GA at baseline. Fleckenstein et al.^67^ and Sunness et al.^68^ reported bilateral GA in 77% and 63%, respectively. In our cohort, 11.6% of patients with nAMD had GA in the fellow eye whereas the aforementioned previous studies report 15% and 27% respectively^67^^,^^68^ These differences may reflect variation in study populations and eligibility criteria including selection toward more symmetric disease phenotypes in our cohort. Furthermore, CARMS grade 5 does not distinguish nAMD alone from nAMD with coexisting atrophic changes. Some patients classified as nAMD may therefore also have had GA. Future studies should investigate whether immune profiles differ between patients progressing from intermediate AMD to nAMD and those developing nAMD in the presence of GA.

This study has limitations that need to be taken into account. Its prospective and cross-sectional design captures only phenotypic T-cell differences at a single time point and does not allow causality or assessment of disease progression. The analysis was limited to a small set age-associated markers and surface phenotypes without functional or mechanistic assays. Subgroup analyses were restricted by sample size, and potential confounding and lifestyle factors may have influenced the results. Our classification by the eye with the more advanced CARMS stage may also obscure bilateral disease burden as patients within the same subgroup may differ in fellow eye-stage. This may be relevant if systemic immune profiles reflect overall bilateral disease burden rather than the phenotype of the more severely affected eye alone. Accordingly, the findings should be considered descriptive and hypothesis generating.

## Conclusions

Our comparison of GA and nAMD with a shared control group revealed age-related T-cell differentiation in late-stage AMD. GA was characterized by increased loss of CD28 on CD8+EM T cells, whereas nAMD showed higher levels of naive CD4+CD56+ and CM T cells relative to GA. These specific AMD subtype differences suggest distinct immune profiles, with CD56+ T cells potentially linked to angiogenic processes in nAMD and their reduction may be associated with GA pathogenesis.

## Supplementary Material

Supplement 1
