# Supplementary material for: Geographic Atrophy in Age-Related Macular Degeneration Is Associated With Distinct T-Cell Differentiation Profile
Source: Invest Ophthalmol Vis Sci. 2026 Nov 2;67(13):3. doi: 10.1167/iovs.67.13.3 (PMC13743819; doi:10.1167/iovs.67.13.3)
Supplement: Supplementary file 1 [file iovs-67-13-3_s001.docx]

### **Supplementary Table 1 - Antibodies used for flowcytometry**

| **Target** | **CD4** | **CD8** | **CD56** | **CCR7** | **CD45ra** | **CD45ro** | **CD27** | **CD28** |
| --- | --- | --- | --- | --- | --- | --- | --- | --- |
| Fluorochrome | PerCP | PE/Cy7 | APC/Cy7 | Brilliant Violet | Pacific Blue | FITC | PE | APC |
| Isotype | IgG2a | IgG1 | IgG1 | IgG2a | IgG2b | IgG2a | IgG1 | IgG1 |
| Clone | #11830 | HIT8a | HCD56 | G043H7 | HI100 | UCHL1 | M-T271 | CD28.2 |
| Manufacturer | R&D Systems | Biolegend | Biolegend | Biolegend | Biolegend | AbD Serotec | Biolegend | Biolegend |
| Negative isotype |  |  |  |  |  |  |  |  |
| Fluorochrome |  |  |  |  |  |  | PE | APC |
| Isotype |  |  |  |  |  |  | IgG1 | IgG1 |
| Clone |  |  |  |  |  |  | MOPC-21 | MOPC-21 |
| Manufacturer |  |  |  |  |  |  | BD Biosciences | Biolegend |

**Supplementary Table 1:** Manufacturers: AbD Serotec, Kidlington, UK; BD Biosciences, Franklin Lakes, NJ, USA; BioLegend, San Diego, CA, USA; R&D Systems, Minneapolis, MN, USA.

*Abbreviations: APC = Allophycocyanin; PE = Phycoerythrin; PerCP: Peridinin chlorophyll; Cy7: Cyanine-7; FITC: Flouresceinisothiocyanat.*

### **Supplementary figure 1 - Lymphocyte gating strategy and expression analysis**

**Supplementary figure 1:** Gating strategy of CD4+ and CD8+ T cells and distribution of the surface markers CD27, CD28 and CD56. Negative control tubes with fluorochrome-matched negative isotypes are also shown.
*Abbreviations: FSC: Forward Scatter, SSC: Side scatter, A: Area, H: Height.*
